# Alternating Magnetic Field-Triggered Switchable Nanofiber Mesh for Cancer Thermo-Chemotherapy

**DOI:** 10.3390/polym10091018

**Published:** 2018-09-13

**Authors:** Eri Niiyama, Koichiro Uto, Chun Man Lee, Kazuma Sakura, Mitsuhiro Ebara

**Affiliations:** 1 International Center for Materials Nanoarchitectonics (WPI-MANA), National Institute for Materials Science (NIMS), Tsukuba, Ibaraki 305-0044, Japan; s1630131@u.tsukuba.ac.jp; 2 Graduate School of Pure and Applied Sciences, University of Tsukuba, Tsukuba, Ibaraki 305-8577, Japan; 3 International Center for Young Scientists (ICYS), National Institute for Materials Science (NIMS), Tsukuba, Ibaraki 305-0044, Japan; UTO.Koichiro@nims.go.jp; 4 Medical Center for Translational Research, Osaka University Hospital, Suita, Osaka 565-0871, Japan; leechan67_1211@hotmail.com (C.M.L.); tg4c_sakura@outlook.jp (K.S.); 5 Graduate School of Industrial Science and Technology, Tokyo University of Science, Katsushika-ku, Tokyo 125-8585, Japan

**Keywords:** fiber, thermo-chemotherapy, drug delivery system, smart polymer, cancer therapy

## Abstract

We have developed a smart anti-cancer fiber mesh that is able to control tumor-killing activity against lung adenocarcinoma precisely. The mesh is capable of carrying large loads of chemotherapeutic drug, paclitaxel (PTX), as well as magnetic nanoparticles (MNPs). The mesh generates heat when the loaded MNPs are activated in an alternating magnetic field (AMF). The mesh is thermo-responsive, so the heat generated can be also used to trigger PTX release from the mesh. An electrospinning method was employed to fabricate the mesh using a copolymer of *N*-isopropylacrylamide and *N*-hydroxymethylacrylamide, the phase transition temperature of which was adjusted to the mild-hyperthermia temperature range around 43 °C. In vitro anti-tumor studies demonstrated that both MNP- and PTX-loaded mesh killed about 66% of cells, whereas only PTX-loaded mesh killed about 43% of cells. In a mouse lung cancer model, the thermo-chemotherapy combo displayed enhanced anti-tumor activity and the systemic toxic effects on mice were eliminated due to local release of the chemotherapeutic agents. The proposed fiber system might provide a blueprint to guide the design of the next generation of local drug delivery systems for safe and effective cancer treatment.

## 1. Introduction

Lung cancer is currently the most common cause of cancer deaths in many countries, including Japan. Non-small cell lung cancer (NSCLC) accounts for 85% of all lung cancer patients, and usually symptoms do not appear until advanced stages [1]. Although cisplatin-based chemotherapy improves survival compared with other conventional chemotherapy regimens, the benefits have been modest [2]. In the last few decades, it has been shown that other chemotherapeutic drugs with novel mechanisms of action, including paclitaxel (PTX), docetaxel, vinorelbine, and gemcitabine, improve survival rates and relieve symptoms in advanced NSCLC cancer patients. Recent studies suggest that using a two-drug combination could be more effective for most lung cancers. For example, the combination of PTX and carboplatin has been a widely used regimen for NSCLC in the USA [3]. More recently, a new medication concept called “dose-dense chemotherapy” has gained much attention as it achieves maximum tumor destruction by increasing the rate of chemotherapy delivery, rather than by increasing dosage. Administration of a weekly dose of chemotherapy, which was previously given every 3 weeks, allows the treatment to interrupt the rapid growth phase of the tumor cells [4]. These facts indicate that “temporal control” of drug administration is significantly important for effective cancer chemotherapy. Moreover, some studies have reported that combined dose-dense therapy with hyperthermia enhances activity against cancerous cells [5,6,7].

Hyperthermia, also called thermotherapy, is a type of cancer treatment in which the body tissue is exposed to high temperatures. This offers a promising prospect for treatment since it causes only minimal adverse effects to adjacent tissue, and thus gives efficient and constructive results. Mammalian cells and tissues are generally thermosensitive, as manifested by protein denaturation and tissue destruction at critically elevated temperature [8]. At less than critical temperatures, the response (recovery or apoptosis) depends on the thermal dose received and the type of cell or tissue involved [9,10]. Whole-body hyperthermia is, however, controversial because of the difficulty in administering and monitoring the thermal dose and an incomplete knowledge of thermal pathophysiology [11,12]. Therefore, local hyperthermia is more favorable in order to overcome the challenges faced with whole-body hyperthermia. However, local hyperthermia techniques suffer from limitations such as poor control of thermal dose and targeting to specific regions. In addition, experimental studies have reported that pre-heat treatment induces a temporary increase in the resistance of cells against subsequent heating [13,14,15]. Therefore, “temporal control” of heating is also required for effective hyperthermia treatment.

**Figure 1:**
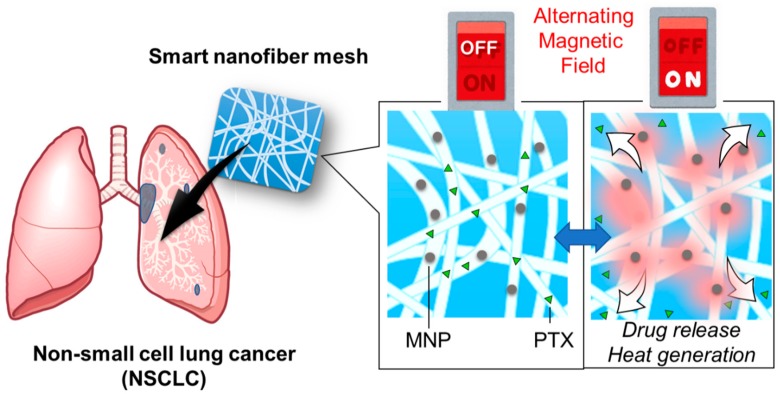
Schematic illustration for on–off switchable temperature-responsive fiber mesh for cancer thermo-chemotherapy. The mesh contains paclitaxel (PTX) and magnetic nanoparticles (MNPs). The mesh releases heat and drug when activated in alternating magnetic field (AMF).

Based on these requirements, we developed a smart anti-cancer fiber mesh that is able to precisely control both the local application of heat and the local administration of chemotherapeutic drugs (Figure 1). We have previously established a fabrication method for on–off switchable fiber platforms for skin cancer treatment in vitro using doxorubicin [16]. PTX is another anti-cancer drug and it has been reported that hyperthermia promotes PTX-induced apoptosis through the activation of caspase-7, increasing the proportion of cells arrested in G2/M (Gap2 phase/ Mitotic phase), thereby reducing the IC_50_ of PTX [17]. Therefore, in this study, we aimed to apply the switchable system to treat NSCLC in vivo using PTX. Although one of the major challenges of PTX in clinical applications is its highly hydrophobic molecular status, the electrospinning method that we used enables fibers to carry large loads of PTX as well as magnetic nanoparticles (MNPs) as a heat source. Another advantage of using the fibrous form is that it can be easily manipulated and implanted because hyperthermia treatment is usually used in conjunction with surgery. A report indicated postoperative intrathoracic chemo-thermotherapy for lung cancer improved 5-year survival rates by eradicating residual tumors after resection and improving the local treatment [18].

## 2. Materials and Methods

### 2.1. Materials

*N*-Isopropylacrylamide (NIPAAm) was purchased from KJ Chemicals Corporation (Tokyo, Japan). 2’2-Azobis(isobutyronitrile) (AIBN) and dimethylformamide (DMF) were obtained from FUJIFILM Wako Pure Chemical Corporation (Osaka, Japan). *N*-Hydroxymethylacryamide (HMAAm), 1,1,1,3,3,3-hexafluoro-2-propanol (HFIP), 1,4-butanediol and paclitaxel (PTX) were purchased from Tokyo Chemical Industry Co., Ltd. (Tokyo, Japan). Iron (III) oxide nanopowder (<50 nm particle size) and tin (II) 2-ethylhexanoate were obtained from Sigma-Aldrich Japan (Tokyo, Japan). RPMI 1640 with L-ln, 5.0 g/L-trypsin/5.3 mmol/L-ethylenediaminetetraacetate solution, antibiotic-antimycotic mixed stock solution, sodium pyruvate solution and MEM non-essential amino acids solution were purchased from Nacalai Tesque, Inc. (Kyoto, Japan). Fetal bovine serum (FBS) was purchased from Tocris Bioscience Inc. (Minneapolis, MN, USA). Alamar blue reagent was obtained from TREK Diagnostics (Cleveland, OH, USA). NCI-H23 (human lung cancer cell line: adenocarcinoma) was purchased from the American Type Culture Collection (Manassas, VA, USA). Female SCID (Severe Combined Immune Deficiency) mice 6 weeks of age were obtained from Charles River Laboratories Japan, Inc. (Yokohama, Japan).

### 2.2. Synthesis of Poly(NIPAAm-co-HMAAm)

As shown in Scheme S1, NIPAAm and HMAAm were copolymerized as described previously [16]. Briefly, NIPAAm (80 mol %), HMAAm (20 mol %) and AIBN (0.01 mol % of total monomer concentration) were dissolved in 20 mL of DMF. The total monomer molar concentration was 50 mmol. The copolymerization was carried out at 60 °C for 20 h after which it was completely degassed via four freeze-thaw cycles. After polymerization, AIBN, unreacted monomers, impurities, and solvent were removed by dialysis against ethanol (FUJIFILM Wako Pure Chemical Corporation, Osaka, Japan) and distilled water for seven days. The dialyzed solutions were lyophilized for four days. The chemical structure of the obtained copolymer was confirmed by ^1^H NMR (JEOL, Tokyo, Japan). The average molecular weight (*M*_n_) and polydispersity index (PDI) of the copolymer were determined by gel permeation chromatography (GPC, JASCO International, Tokyo, Japan) using DMF with lithium bromide (LiBr, 10 mM) (Tosho Corporation, Tokyo, Japan) as an eluent sample. The temperature-dependent transmittance change of the copolymer in phosphate buffered saline (PBS) (Nakalai Tesque, Kyoto, Japan) (pH = 7.4, 0.1 *w*/*v* %) was measured by UV-Visible spectrophotometer (JASCO corporation, Tokyo, Japan) with a heating rate of 1.0 °C/min. The lower critical solution temperature (LCST) of the copolymer was defined as the temperature at 50% of transmittance.

### 2.3. Fabrication of Fiber Meshes

Electrospinning solution was prepared by dissolving poly(NIPAAm-*co*-HMAAm) in HFIP (20 *w*/*v* %). MNPs and PTX were dissolved in the electrospinning solution at concentrations of 30 *w*/*w* % and 0.75 *w*/*w* %, respectively. The solution was electrospun into fibers using an applied voltage of 20 kV with a 13 cm separation of the needle and collector plate (Nanon-01A, MECC Co., Ltd., Fukuoka, Japan) [16]. The flow rate was set to 1.0 mL/h. Electrospun fibers were put in an oven (TOKYO RIKAKIKAI Co., Ltd, Tokyo, Japan) at 130 °C for 24 h to remove organic solvents. This process also enables the fibers to crosslink polymer chains due to self-condensation reaction of methylol group in HMAAm upon heating [16]. The morphology of fibers was observed by a scanning electron microscope (SEM; SU8000, Hitachi High-Technologies Corporation, Tokyo, Japan) using secondary electrons (SE) after Pt coating of the fiber surface. Fiber diameter was calculated from obtained SEM image using ImageJ software. Energy dispersive X-ray spectroscopy (FE-SEM SU8000 EDX, Hitachi High-Technologies Corporation, Tokyo, Japan) Mapping (with Bruker QUANTAX EDS for SEM, 5 kV) for iron in the MNPs was used to observe the localization of the MNPs within the fibers. A transmission electron microscope (TEM; JEM-1010, JEOL Ltd., Tokyo, Japan) was used for confirmation of embedded MNPs inside the fibers. For TEM sample preparation, the fiber was directly electrospun onto a copper grid (JEOL Ltd., Tokyo, Japan). Embedded MNPs inside the fibers were also evaluated via the weight loss as a function of temperature by Thermogravimetry-Differential Thermal Analysis (TG/DTA) (EXSTAR6000 TG/DTA, SII Nanotechnology, Tokyo, Japan). Crosslinking between methylol groups of HMAAm within the fiber was confirmed based on the disappearance of absorbance at 1050 cm^−1^ by Fourier Transform Infrared Spectroscopy (IRPrestige-21, Simazu, Kyoto, Japan) by KBr method. All analyses were carried out after removal of residual solvent in the fibers by vacuum drying (ULVAC KIKO, Inc., Miyazaki, Japan).

### 2.4. Evaluation of Thermo-Responsive Swelling/Deswelling Behavior

To characterize swelling/de-swelling behavior of the fiber meshes, the weight increase/decrease was measured, respectively, after immersing in water at different temperatures. First, dried fiber (approximately 4.2 mg) was immersed in 1 mL of pure water at 25 °C and equilibrated for 15 min. The fiber weight was measured after removing excess water around the fiber mesh (swollen state). Then, the fiber was heated to 45 °C with a heater for 15 min and the weight was measured again (dehydrated state). To observe the swelling/de-swelling behavior of individual fibers more precisely, atomic force microscopy (AFM; MFP-3D origin, Oxford Instruments plc, Oxon, UK) was used. The fiber was directly electrospun on cover glass and allowed to swell in 1 mL of water at 25 °C. The sample was then heated to 50 °C for 30 min.

### 2.5. Heating Profiles

The heating profiles of MNP-loaded fibers were investigated by placing the fiber mesh in the center of a copper coil and applying an alternating magnetic field (AMF, HOSHOT2, Alonics Co., Ltd., Tokyo, Japan). Heat was generated by the AMF (480 A, 192 kHz frequency, 362 W). The heating profiles were obtained by taking photos every 30 or 60 s using FLIR thermo camera (CPA-E6, FLIR systems Japan K.K., Tokyo, Japan).

### 2.6. Alternating Magnetic Field (AMF)-Responsive Drug Release

The MNP and PTX-loaded fiber mesh (40 mg) was first immersed in 500 μL of PBS at room temperature for 15 min. After reaching equilibrium state, the sample was placed in a copper coil. Then AMF was applied for 15 min and released PTX was collected. The released amount of PTX was quantified by an ultraviolet-visible (UV-Vis) spectrophotometer (V-650 spectrophotometer, Jasco, Tokyo, Japan). This process was repeated 8 times. The cumulative released PTX was calculated using the following equation:

Cumulated released PTX (%) = (*D*_released at X cycle_/*D*_total_) × 100 (1)

where *D*_released at X cycle_ is the cumulative amounts of released PTX at X cycle of AMF irradiation and *D*_total_ is the total amount of incorporated PTX in the fibers.

### 2.7. Long-Term Drug Release

To observe the long-term drug release behavior, the MNP- and PTX-loaded fiber mesh (40 mg) was immersed in 5 mL of PBS at 25, 37 or 45 °C. Two milliliters of released PTX in PBS was collected and 2 mL of fresh PBS was added to each sample. The released amount of PTX was quantified as identified above.

### 2.8. Anti-Tumor Activities In Vitro

The anti-tumor effects of all fiber meshes were evaluated using NCI-H23 (human lung adenocarcinoma) cells. Cells were purchased from American Type Culture Collection (Manassas, VA, USA). Cells were cultured using RPMI1640 supplemented with 10% FBS, 1% antibiotic-antimycotic mixed stock solution, sodium pyruvate solution, and MEM non-essential amino acids solution. Cells were maintained at 37 °C in 5% CO_2_ during culturing. NCI-H23 cells were seeded on 35 mm dish (AGC TECHNO GLASS Co., Ltd., Shizuoka, Japan) for 24 h before co-culturing them with the meshes. The samples were exposed to AMF irradiation for 15 min and then cultured for another 24 h at 37 °C. Alamar blue assay reagent (10% against medium) was then added to each dish and incubated for 1 hour at 37 °C, according to a protocol. Cell number was calculated from fluorescent intensity measured by fluorescent plate reader (PerkinElmer Co., Ltd., Kanagawa, Japan).

### 2.9. Animal Studies

A lung tumor-bearing mouse model was used in this study. NCI-H23 cells (10^8^ cells/mL) were injected subcutaneously into the back of CB17/SCID mice. The fiber meshes were implanted adjacent to the tumor region of the mice after the tumors reached a size of 100 mm^3^. The size of the implanted fibers was around 8 mm^2^, 40 mg/piece (0.3 mg PTX/piece). The control mouse received no treatment. Each mouse with fiber was subjected to AMF once a week for 15 min for 2 months to apply heat treatment to the tumor. After 2 months, the tumors and transplanted fibers were removed from the mouse. Tumor size was measured with a ruler and death of cancer cells was evaluated from the calculated tumor volume change over 2 months. Five mice were used for each experiment. All mice were euthanized before terminal symptoms appeared. All animal care and experimental procedures were approved by the Experimental Animal Administration Committee of National Institute for Materials Science (36-2013-2).

## 3. Results and Discussion

### 3.1. Temperature-Responsive Fiber Mesh

**Figure 2:**
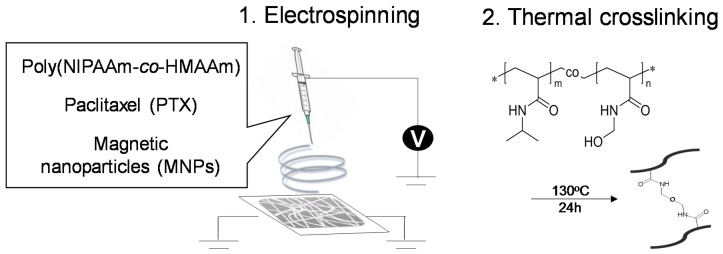
Fabrication of thermally-crosslinkable temperature-responsive fiber mesh by electrospinning and thermal curing processes by self-condensation.

**Figure 3:**
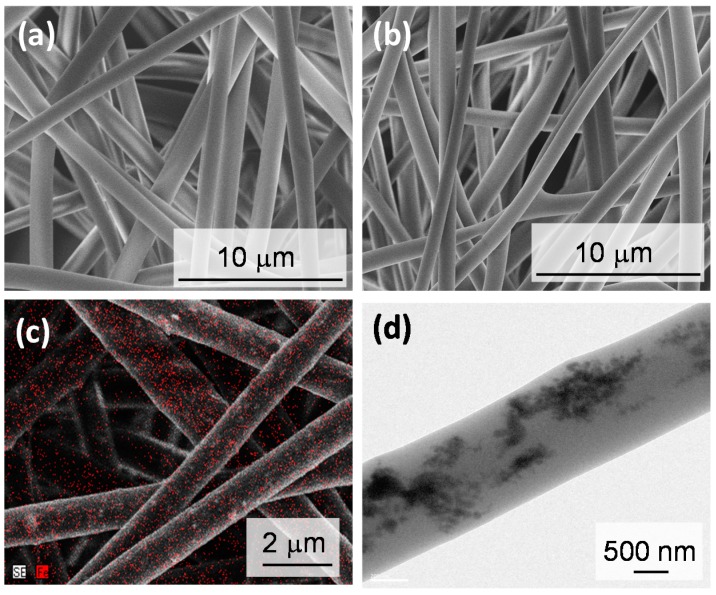
Scanning electron microscope (SEM) images of the fiber mesh before (**a**) and after (**b**) thermal crosslinking. (**c**) energy dispersive X-ray spectroscopy (EDX) image and (**d**) transmission electron microscope (TEM) image of MNP-loaded fibers (red: Fe element).

Temperature-responsive fiber meshes with switchable property were fabricated by electrospinning the copolymer of NIPAAm and HMAAm (Figure 2). Hydrophilic monomer HMAAm was used to adjust the lower critical solution temperature (LCST) of the copolymer to around hyperthermia temperature (~45 °C). The HMAAm content in the copolymer was determined to be 21 mol % calculated from comparison with isopropyl group (1H) of the NIPAAm and methylol group (2H) of HMAAm by ^1^H NMR (Figure S1). The corresponding LCST value was 43 °C (Figure S2). The LCST was set slightly below 45 °C. In this study, we synthesized the copolymer with relatively large molecular weight (M_n_ ~50 k) (Table S1), because lower molecular weight may result in the formation of beads or particles due to insufficient molecular chain entanglement during the electrospinning process. As the molecular weight increases, the fiber morphology changes from a bead form to fine fibers. In our previous report [19], PNIPAAm with M_n_ = 10 k formed a bead-like structure at 1.0–10 *w*/*v* % of solution concentration. In the case of PNIPAAm with relatively higher molecular weight, fiber was electrospun on a lower solution concentration such as 1.0–3.0 *w*/*v* %. In this study, we optimized the fiber fabrication conditions as follows: 22 gauge needle, 20 wt % of polymer concentration, 20 kV of applied voltage, and 13 cm gap between collector and needle. As shown in Figure 3a, bead-free, smooth, and uniform fibers were formed with an average diameter of 1.18 ± 0.03 μm.

Another reason for the introduction of HMAAm units is to crosslink polymer chains. One of the challenges in the development of NIPAAm-based fibers is the stability in aqueous media. For example, electrospun fibers from NIPAAm homopolymer disperse in water easily [19]. Although physical crosslinking can be accomplished by hydrophobic interactions [20,21], chemical crosslinking is more favorable. With this background, thermal crosslinking was carried out at 130 °C for 24 h to cure the methylol groups in HMAAm. The two methylol groups formed a methylene bridge. The successful formation was confirmed by observing the disappearance of the peaks corresponding to methylol groups in the attenuated total reflection Fourier-transform infrared spectroscopy (ATR-FTIR) spectra (Figure S3). The peaks at 1650 and 1550 cm^−1^ were assigned to amide I (C=O stretching) and amide II (N–H bending) of the copolymer, respectively. The broad absorption band observed around 3430 cm^−1^ was assigned to the N–H stretching of amide groups in the copolymer. The peaks at 1370–1390 and 2980 cm^−1^ were assigned to the stretching modes of the –CH(CH_3_)_2_ and –(CH_3_)_2_ groups in NIPAAm, respectively. The peaks at 1050, 1230, and 3300 cm^−1^ were assigned to the C–O–H stretching, C–O–H bending, and O–H stretching modes in HMAAm, respectively. Unfortunately, quantitative analysis of crosslinking density was difficult from the obtained ATR-FTIR spectra in this study. However, the signal intensity of methylol groups gradually decreased with increasing thermal treatment time. Figure 3b shows the SEM image for the fibers after thermal crosslinking. Fibrous structures were maintained and the average diameter was 1.07 ± 0.03 μm.

Figure 3c shows EDX mapping of the fiber mesh containing MNPs. Red dots (Fe element) were homogeneously distributed in the entire mesh. The TEM image also revealed that the MNPs were successfully incorporated within the fiber (Figure 3d). From TGA, about 15 *w*/*w* % of MNPs was incorporated (Figure S4). In general, the concentration of MNPs is an important parameter to obtain well-defined fibers as the interaction between polymers and MNPs affects the viscosity of the mixing solution. In case of fibers with more than 60 wt % of MNPs, for example, the aggregates affected fiber morphologies (Figure S5). The other concern is that aggregates may have different effects on converted thermal energy from magnetic energy.

### 3.2. AMF-Responsive Heat Generation

**Figure 4:**
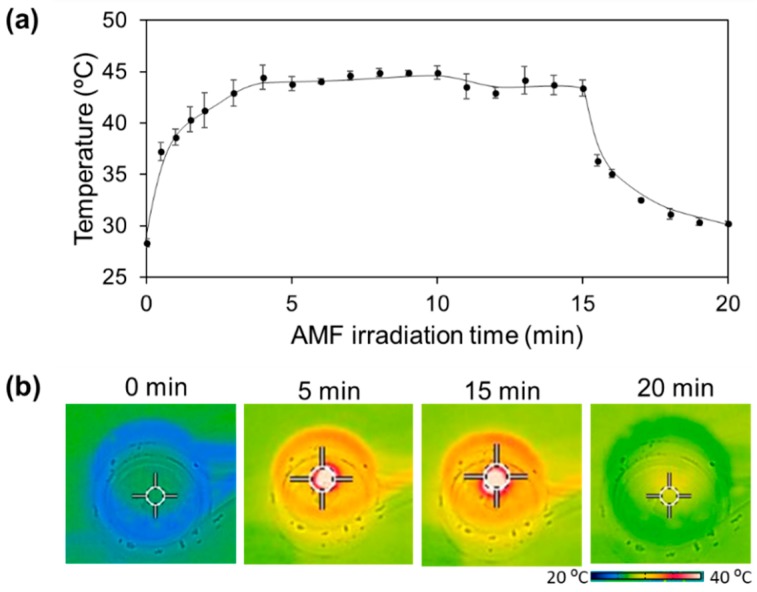
(**a**) AMF-responsive heating/cooling profiles of the MNPs-loaded fiber mesh. AMF was irradiated for 15 min and then turned off. (**b**) The infrared thermal images of the MNPs-loaded fiber mesh in a copper coil.

In hyperthermia treatment, radiofrequency, microwaves, and focused ultrasound waves are commonly used methods for heat generation [22]. The use of MNPs as a heat source, known as magnetic hyperthermia, is the most recent technology to deliver heat to tumor cells. The heat generation is based on the application of an AMF to the MNPs. The remote action of the AMF to produce heat makes magnetic hyperthermia an attractive treatment for eliminating non-accessible, deep tumors. Figure 4a depicts the time-dependent temperature changes of the fiber mesh with 12 mg of MNPs, determined by the infrared thermal images. The fiber temperature increased from 27.6 to 43.1 °C within 5 min upon AMF irradiation (Figure S6). The infrared thermal images captured during these experiments show the temperature distribution was even in the mesh (Figure 4b). The heating profiles are also dependent on the concentration of MNPs, AMF power, and coil diameter. For example, only a very small increase in temperature was observed for the sample with 0.9 mg of MNPs, while the sample with 45 mg of MNPs was heated above 50 °C within 40 s (data not shown). The heating ability was also controllable by AMF frequency since the transition radiation of electron energy levels of MNPs and the change of spin state lead to the enhancement of Brownian and Néel relaxation [23]. This switchable ability of the fiber mesh is very appealing because current hyperthermia treatment has many limitations such as thermal dose control. Also, it has been reported that pre-heat treatment induces a temporary increase in the resistance of cells against subsequent heating [13,14,15]. Therefore, on–off switchable control of heating using the fiber mesh can improve the thermo killing effects.

### 3.3. AMF-Fesponsive Drug Release

**Figure 5:**
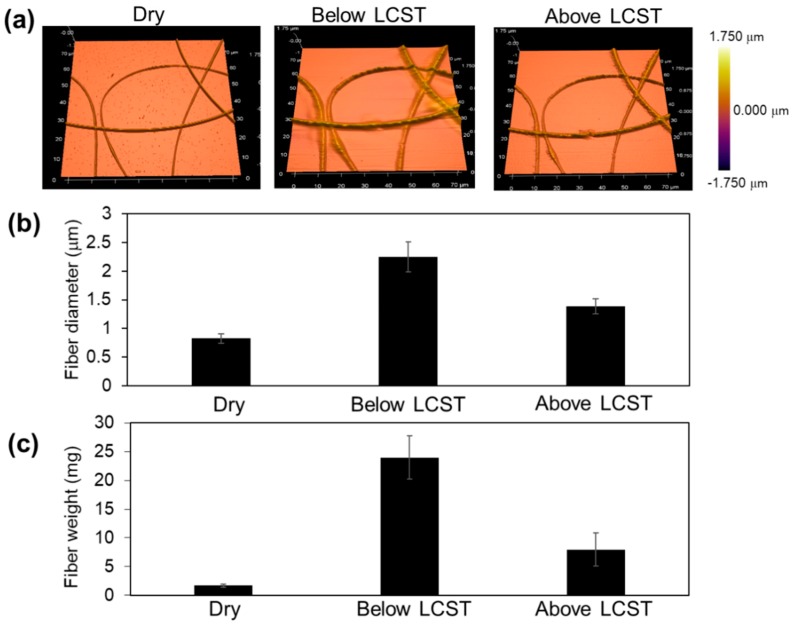
(**a**) AFM images, (**b**) the corresponding fiber diameters, and (**c**) fiber weights at dry state (left), at swollen state in water below the LCST (middle), and at dehydrated state above the LCST (right).

In this study, we designed a temperature-responsive fiber mesh to demonstrate on-off PTX release in response to external AMF irradiation, which leads to self-generated heat from the incorporated MNPs to induce the de-swelling of polymer networks in the fiber. Therefore, temperature-dependent switchable changes in the swelling ratio of poly(NIPAAm-*co*-HMAAm) mesh were evaluated. Figure 5a shows AFM images of the fiber morphology. In a dry state (left), the fiber diameter was found to be 0.83 μm (Figure 5). After incubation in water below the LCST, the fiber swelled to achieve a diameter of 2.2 μm (middle image). On the other hand, the diameter was 1.4 μm when it was heated above the LCST (right image). Of particular importance is that the crosslinked fibers did not dissolve in water and the fibrous structures were still visible both below and above the LCST. The weight changes of the fiber mesh at different temperatures were also evaluated. The original fiber weight was 1.7 ± 0.3 mg (Figure 5c). After incubation in water below and above the LCST, the fiber weight became 24.0 ± 2.2 and 8.0 ± 1.3 mg, respectively. In other words, 1.7 mg of fiber can absorb 22.3 mg of water below the LCST, while above the LCST it can release 6.3 mg of water, indicating that 28% of water in the fiber was squeezed out by heating. This value is not necessarily large when compared with conventional NIPAAm bulk hydrogels. In case of fibrous materials, both inter and intra-fiber water have to be considered due to the porous structure when the swelling/de-swelling ratio is measured. Therefore, careful removal of the excess water is always a big challenge.

**Figure 6:**
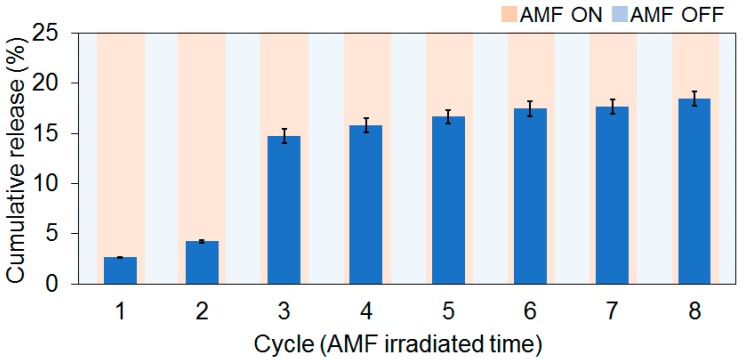
PTX release from the fiber in response to AMF irradiation.

Next, AMF-triggered PTX release from the fiber was investigated. The fiber meshes were equilibrated for 15 min at room temperature and then AMF was irradiated for 15 min. The AMF irradiation cycles were repeated 8 times in total. The cumulative release of PTX is shown in Figure 6. About 8, 13, and 44 μg/mL of PTX were released at the 1st, 2nd, and 3rd cycles, respectively. PTX release behavior during the first 3 cycles was not stable. This is plausibly owing to the unique morphology of fibrous material as mentioned. After the 4th cycle, constant release (average 2 μg/mL/cycle) was observed and the total released amount of PTX reached to approximately 37 μg/mL (0.043 nM), which corresponds to 18% of the loaded PTX in the fiber (average cumulated released PTX per cycle was 6.9 μg). This value is much lower than the previously reported IC_50_ value of PTX for NSCLC (H358; IC_50_ 110 nM [24]. However, our strategy aims at local drug delivery instead of the whole body. Therefore, we believe that higher dosage is not necessary.

PTX is one of the most difficult drugs to control in terms of its release properties because it hardly dissolves in aqueous media. Figure S7 shows PTX release profiles at constant temperatures of 25, 37, or 43 °C. Regardless of temperature, little PTX (less than 3%) was released within 2 weeks. Despite that, an average of 6.9 μg/mL of PTX during the 8th cycle of the 15-min AMF irradiation was squeezed out from the fiber. This fact indicates that dynamic dehydration of polymer chains caused by self-heating of the incorporated MNPs is very effective to release PTX from the fiber. Furthermore, this on–off switchable drug release is also useful from the viewpoint of dose-dense treatment in which frequent drug administration with low drug dose is more effective than dose-escalated administration [25]. Katsumata et al. compared PTX administration paradigm of a weekly dose with the conventional one once every three week administration, for treatment of advanced epithelial ovarian cancer, and found that weekly administration was more effective [4]. This dose-dense concept has been applied for several different kinds of cancer such as primary and advanced breast cancer [25,26,27,28], ovarian cancer [28] and also NSCLC [29]. Thus, everything considered, our on-off switchable fiber system is very attractive for controllable chemotherapy.

### 3.4. Anti-Tumor Effects

**Figure 7:**
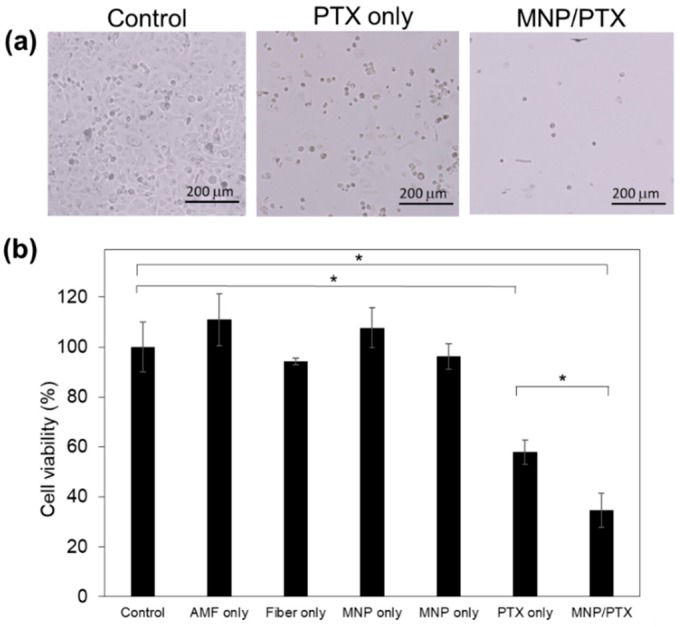
Anticancer effect of PTX on NCI-H23 cells. AMF was applied for 15 min. (**a**) Phase contrast microscopic images of cell treated with the fiber with PTX and MNP/PTX. (**b**) Cell viability after the treatment (*N* = 3, * *p* < 0.05).

Anti-tumor effects of the fiber mesh were evaluated in vitro and in vivo. NCI-H23 cells and the mesh were co-cultured for 24 h and the dish was exposed to AMF once every 15 min. Figure 7 compares cell viability after the treatment. Neither nanofiber itself (fiber only) nor AMF itself (AMF only) did not affect cell viability. In addition, the hyperthermia effect itself (MNP only) showed a minor anti-cancer effect for a short duration of AMF application. The fiber mesh containing only PTX killed 43% of cells, whereas the mesh containing both PTX and MNPs killed 66%. This is because the released PTX from the fiber would induce strong anticancer effects due to a synergistic effect in combination with hyperthermia. If hyperthermia enhanced the anti-tumor effect of PTX, these observations become consistent with the fact that a low concentration of PTX is enough to suppress spindle microtubule dynamics in cancer cells at higher temperature, which causes a disorganization of the microtubule system.

**Figure 8:**
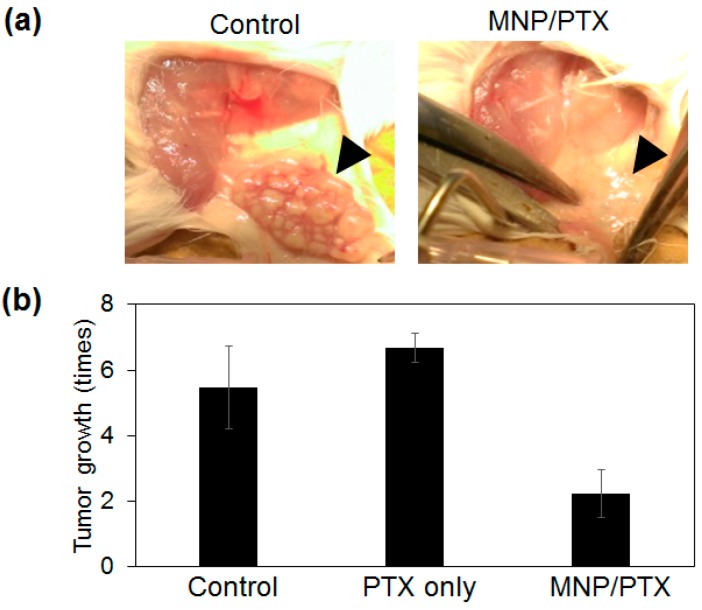
(**a**) Photographs of tumors after the treatment with (right) and without (left) fiber. (**b**) Tumor growth ratio after the treatment with fiber containing PTX and MNP/PTX.

The in vivo anti-cancer effect was evaluated using a tumor-bearing mouse. AMF was irradiated for 15 min per week. Figure 8 compares the tumor growth after 8 cycles of AMF irradiation (2 months). For control groups, the size of the tumor increased more than 5 times within 2 months. Even only PTX-containing fiber mesh could not suppress the tumor growth in this study, although this may be improved with increased concentrations of PTX. On the other hand, the fiber containing both PTX and MNPs significantly suppressed the tumor growth. As expected, the anticancer effect of the fiber containing only MNPs was not significant (data not shown). This tumor growth-suppressing effect was significant compared with a previous report where an injection of PTX per week (weekly 1 mg/kg) led to tumor growth from 200 to 1210 mm^3^ in 8 weeks [24]. Compared with this data, our MNP/PTX fiber inhibited tumor growth by a factor of 2.2 with a lower drug dose (weekly 0.37 mg/kg). Furthermore, the median lethal dose (LD_50_) of i.v. PTX in male Sprague–Dawley rats was reported to be 8.3 mg/kg [30]. Taken together, our results indicate that the thermo-chemotherapy combo synergistically enhanced the anti-tumor activity and systemic toxic effects on the mice were eliminated due to the precise release of the chemotherapeutic agents.

## 4. Conclusions

In conclusion, this study demonstrates the efficiency of a combined thermo-chemotherapeutic system against lung cancer models using switchable fiber mesh. The fiber mesh was successfully fabricated by an electrospinning method with temperature-responsive polymer, PTX, and MNPs. By chemical crosslinking, the fiber mesh showed reversible changes in swelling ratio in response to AMF irradiation. The AMF-triggered heat generation, followed by PTX release from the fiber, was observed because the MNPs within the fiber generated localized heat, causing dehydration and shrinking of the fiber. In vivo experiments demonstrated that MNP/PTX fiber inhibited tumor growth with much lower PTX concentration than a lethal dose. These results proved that the switchable fiber platform might serve as a safe and efficient thermo-chemotherapeutic combo for cancer therapy.

## Supplementary Materials

The following are available online at http://www.mdpi.com/2073-4360/10/9/1018/s1, Scheme S1: Copolymerization of poly(NIPAAm-*co*-HMAAm), Figure S1: ^1^H NMR spectrum of poly(NIPAAm-*co*-HMAAm), Figure S2: Temperature-dependent transmittance changes of PNIPAAm and poly(NIPAAm-*co*-HMAAm) solution, Figure S3: FTIR spectra of poly(NIPAAm-*co*-HMAAM) with different crosslinking time (0–72 h). Peak at 1050 and 3300 cm^−1^ are assigned to the stretching of C–O–H and –OH groups in HMAAm, respectively, Figure S4: Thermal studies showing the TG curves for fiber only and MNP, Figure S5: TEM observation of fiber with 60 wt % of MNPs. A scale bar is 500 nm, Figure S6: IR thermal images of the MNPs-incorporated fiber mesh in AMF. The AMF was turned on at time 0-min and turned off at time 15-min, Figure S7: Long term drug release from the fiber mesh at 25 °C, 37 °C, and 43 °C, Table S1: Characteristic data of poly(NIPAAm-*co*-HMAAm).

## References

[1] Oser M.G., Niederst M.J., Sequist L.V., Engelman J.A. (2015). Transformation from non-small-cell lung cancer to small-cell lung cancer: Molecular drivers and cells of origin. Lancet Oncol..

[2] Ohguri T., Imada H., Narisada H., Yahara K., Morioka T., Nakano K., Miyaguni Y., Korogi Y. (2009). Systemic chemotherapy using paclitaxel and carboplatin plus regional hyperthermia and hyperbaric oxygen treatment for non-small cell lung cancer with multiple pulmonary metastases: Preliminary results. Int. J. Hyperth..

[3] Bunn P.A., Kelly K. (1998). New chemotherapeutic agents prolong survival and improve quality of life in non-small cell lung cancer: A review of the literature and future directions. Clin. Cancer Res..

[4] Katsumata N., Yasuda M., Isonishi S., Takahashi F., Michimae H., Kimura E., Aoki D., Jobo T., Kodama S., Terauchi F. (2013). Long-term results of dose-dense paclitaxel and carboplatin versus conventional paclitaxel and carboplatin for treatment of advanced epithelial ovarian, fallopian tube, or primary peritoneal cancer (JGOG 3016): A randomised, controlled, open-label trial. Lancet Oncol..

[5] Zwischenberger J.B., Vertrees R.A., Woodson L.C., Bedell E.A., Alpard S.K., McQuitty C.K., Chernin J.M. (2001). Percutaneous venovenous perfusion-induced systemic hyperthermia for advanced non-small cell lung cancer: Initial clinical experience. Ann. Thorac. Surg..

[6] Lyman G.H., Barron R.L., Natoli J.L., Miller R.M. (2012). Systematic review of efficacy of dose-dense versus non-dose-dense chemotherapy in breast cancer, non-Hodgkin lymphoma, and non-small cell lung cancer. Crit. Rev. Oncol. Hematol..

[7] Miller A.A., Wang X.F., Gu L., Hoffman P., Khatri J., Dunphy F., Edelman M.J., Bolger M., Vokes E.E., Green M.R. (2008). Phase II randomized study of dose-dense docetaxel and cisplatin every 2 weeks with pegfilgrastim and darbepoetin alfa with and without the chemoprotector BNP7787 in patients with advanced non-small cell lung cancer (CALGB 30303). J. Thorac. Oncol..

[8] Harmon B.V., Corder A.M., Collins R.J., Gobe G.C., Allen J., Allan D.J., Kerr J.F.R. (1990). Cell-death induced in a murine mastocytoma by 42–47 degrees hating in vitro evidence that the form of death changes from apoptosis to necrosis above a critical heat load. Int. J. Radiat. Biol..

[9] Saparfto S.A., Dewey W.C. (1984). Thermal dose determination in cancer therapy. Int. J. Radiat. Oncol. Biol. Phys..

[10] Roizin-Towle L., Pirro J.P. (1991). The response of human and rodent cells to hyperthermia. Int. J. Radiat. Oncol. Biol. Phys..

[11] Gerweck L.E. (1985). Hyperthermia in cancer therapy: The biological basis and unresolved questions. Cancer Res..

[12] Li G.C. (1984). Thermal biology and physiolosy in clinical hyperthermia—Current status and future-needs. Cancer Res..

[13] Carretero M.T., Carmona M.J., Díez J.L. (1991). Thermotolerance and heat shock proteins in chironomus. J. Insect Physiol..

[14] Jognston R.N., Kucey B.L. (1988). Competitive inhibition of hsp70 gene expression causes thermosensitivity. Science.

[15] Rordorf G., Koroshetz W.J., Bonventre J.V. (1991). Heat shock protects cultured neurons from glutamate toxicity. Neuron.

[16] Kim Y.J., Ebara M., Aoyagi T. (2013). A smart hyperthermia nanofiber with switchable drug release for inducing cancer apoptosis. Adv. Funct. Mater..

[17] Lin Y., Liu Z., Li Y., Liao X., Liao S., Cen S., Yang L., Wei J., Hu X. (2013). Short-term hyperthermia promotes the sensitivity of MCF-7 human breast cancer cells to paclitaxel. Biol. Pharm. Bull.

[18] Kodama K., Doi O., Higashiyama M., Yokouchi H., Tafsuta M. (1993). Long-term results of postoperative intrathoracic chemo-thermotherapy for lung cancer with pleural dissemination. Cancer.

[19] Maeda T., Kim Y.J., Aoyagi T., Ebara M. (2017). The design of temperature-responsive nanofiber meshes for cell storage applications. Fibers.

[20] Gulyuz U., Okay O. (2015). Self-healing poly(*N*-isopropylacrylamide) hydrogels. Eur. Polym. J..

[21] Erbil C., Toz E., Akdemir O., Uyanik N., Clarson S.J., Owen M.J., Smith S.D., VanDyke M.E. (2010). An investigation into the influence of crosslinker type and solvent composition on physical properties and phase transition behavior of poly(*N*-isopropylacrylamide) hydrogels. Advances in Silicones and Silicone-Modified Materials.

[22] Chicheł A., Skowronek J., Kubaszewska M., Kanikowski M. (2007). Hyperthermia-description of a method and a review of clinical applications. Rep. Pract. Oncol. Radiother..

[23] Deatsch A.E., Evans B.A. (2014). Heating efficiency in magnetic nanoparticle hyperthermia. J. Mgn. Mgn. Mater..

[24] Nguyen D.M., Lorang D., Chen G.A., Stewart IV J.H., Tabibi E., Schrump D.S. (2001). Enhancement of paclitaxel-mediated cytotoxicity in lung cancer cells by 17-allylamino geldanamycin: In vitro and in vivo analysis. Ann. Thorac. Surg..

[25] Fornier M., Norton L. (2005). Dose-dense adjuvant chemotherapy for primary breast cancer. Breast Cancer Res..

[26] Kellokumpu-Lethinen P., Tuunanen T., Asola R., Elomaa L., Heikkinen M., Kokko L., Jarvenpaa R., Lehtinen I., Maiche A., Kaleva-Kerola J. (2013). Weekly paclitaxel-an effective treatment for advanced breast cancer. Anticancer Res..

[27] Hudis C., Seidman A., Baselga J., Raptis G., Lebwohl D., Gilewski T., Moynahan M., Sklarin N., Fennelly D., Crown J.P.A. (1999). Sequential dose-dense doxorubicin, paclitaxel, and cyclophosphamide for resectable high-risk breast cancer: Feasibility and efficacy. J. Clin. Oncol..

[28] Seidman A.D., Hudis C.A., Albanel J., Tong W., Tepler I., Currie V., Moynahan M.E., Theodoulou M., Gollub M., Baselga J. (1998). Dose-dense therapy with weekly 1-hour paclitaxel infusions in the treatment of metastatic breast cancer. J. Clin. Oncol..

[29] Petty W.J., Laudadio J., Brautnick L., Lovato J., Dotson T., Streer N.P., Weaver K.E., Miller A.A. (2013). Phase II trial of dose-dense chemotherapy followed by dose-intense erlotinib for patients with newly diagnosed metastatic non-small cell lung cancer. Int. J. Oncol..

[30] Kim S.C., Kim D.W., Shim Y.H., Bang J.S., Oha H.S., Kim S.W., Seo M.H. (2001). In vivo evaluation of polymeric micellar paclitaxel formulation: Toxicity and efficacy. J. Control. Release.

