# Supplementary material for: Alternating Magnetic Field-Triggered Switchable Nanofiber Mesh for Cancer Thermo-Chemotherapy
Source: Polymers (Basel). 2018 Sep 13;10(9):1018. doi: 10.3390/polym10091018 (PMC6404069; doi:10.3390/polym10091018)
Supplement: Supplementary file 1 [file polymers-10-01018-s001.pdf]

Supplementary Information

Alternating Magnetic Field-triggered Switchable Nanofiber Mesh for Cancer Thermo-chemotherapy

Eri Niiyama[1,2], Koichiro Uto[3], Chun Man Lee[4], Kazuma Sakura[4], and Mitsuhiro Ebara[1,2,5*]

[1] International Center for Materials Nanoarchitectonics (WPI-MANA), National Institute for Materials Science (NIMS), Tsukuba, Ibaraki 305-0044, Japan
[2] Graduate School of Pure and Applied Sciences, University of Tsukuba, Tsukuba, Ibaraki 305-8577, Japan
[3] International Center for Young Scientists (ICYS), National Institute for Materials Science (NIMS), Tsukuba, Ibaraki 305-0044, Japan
[4] Medical Center for Translational Research, Osaka University Hospital, Suita, Osaka 565-0871, Japan.
[5] Graduate School of Industrial Science and Technology, Tokyo University of Science, Katsushika-ku, Tokyo 125-8585, Japan.

Table S1. Characteristic data of poly(NIPAAm-*co*-HMAAm).

Feed (mol%)		Contents (mol%) [1]		Mn [2]	Mw [2]	Mw/Mnm [2]
NIPAAm	HMAAm	NIPAAm	HMAAm			
80	20	79	21	4.99×10^4	1.39×10^5	2.78

[1] Measured by ^{1}H NMR. [2] Measured by GPC.

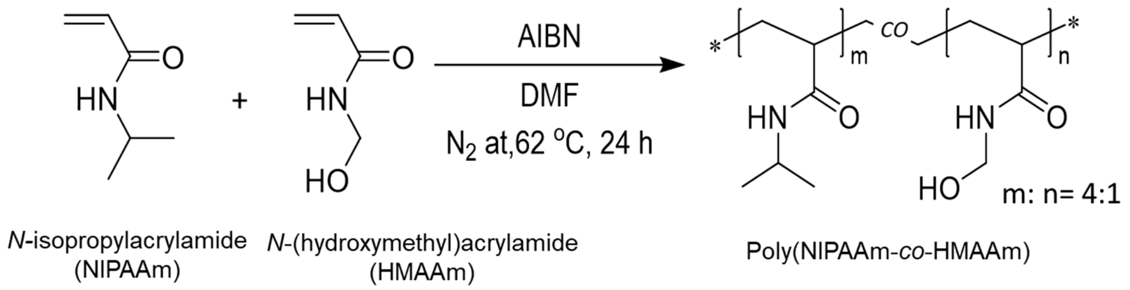

Scheme S1. Copolymerization of poly(NIPAAm-*co*-HMAAm).

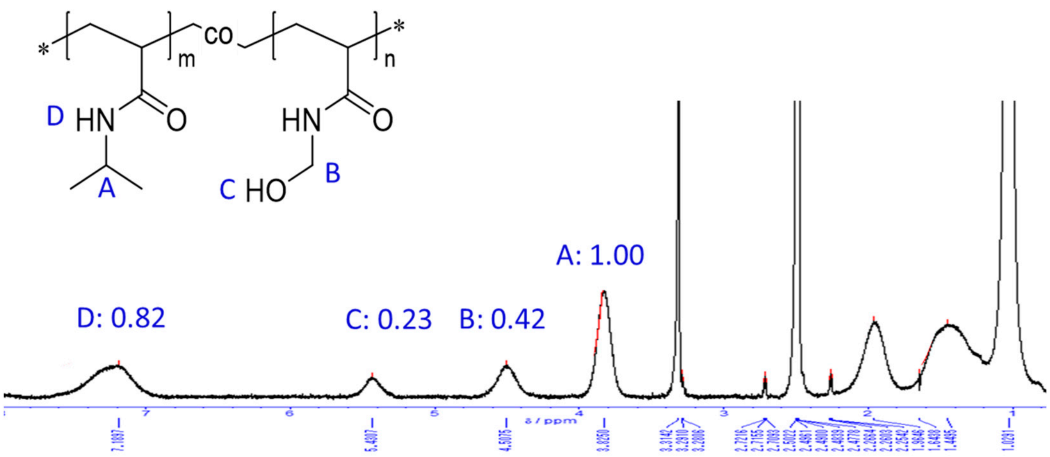

Figure S1. ^{1}H NMR spectrum of poly(NIPAAm-*co*-HMAAm).

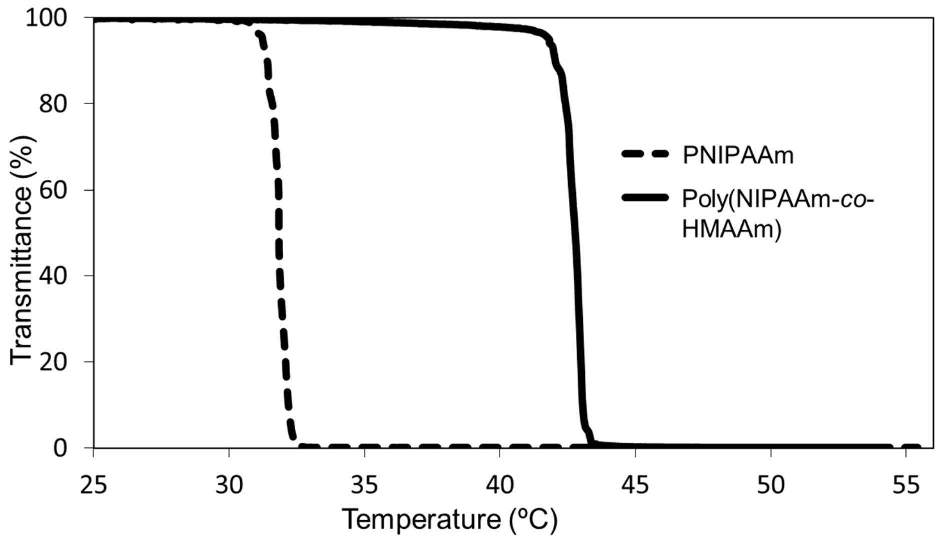

Figure S2. Temperature-dependent transmittance changes of PNIPAAm and poly(NIPAAm-*co*-HMAAm) solution.

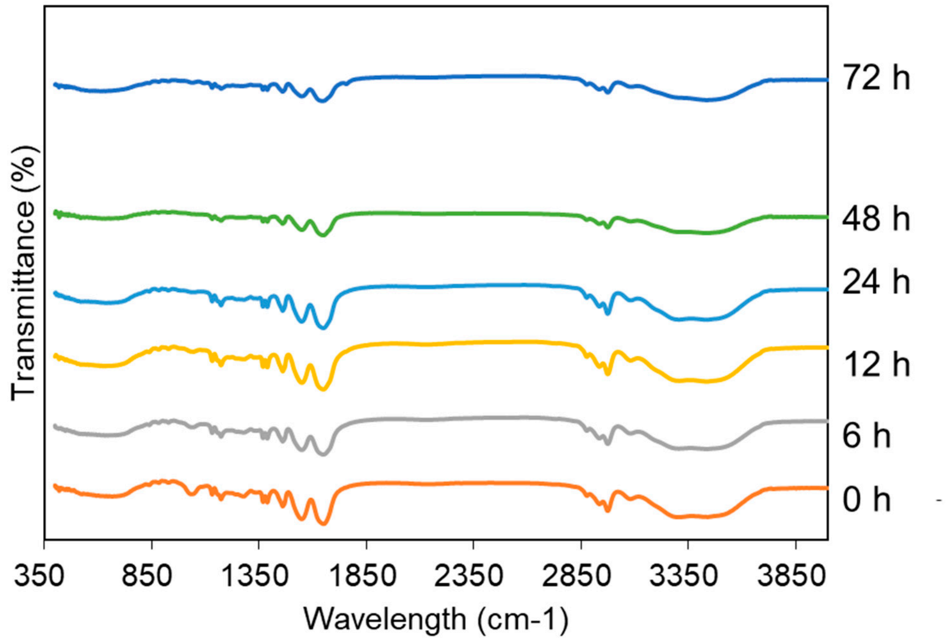

Figure S3. FTIR spectra of poly(NIPAAm-*co*-HMAAM) with different crosslinking time (0-72 h). Peak at 1050 and 3300 cm^{-1} are assigned to the stretching of C–O–H and –OH groups in HMAAm, respectively.

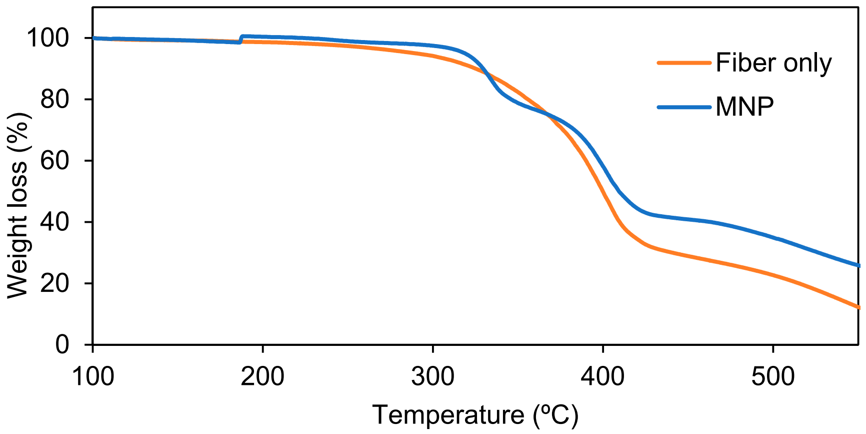

Figure S4. Thermal studies showing the TG curves for fiber only and MNP.

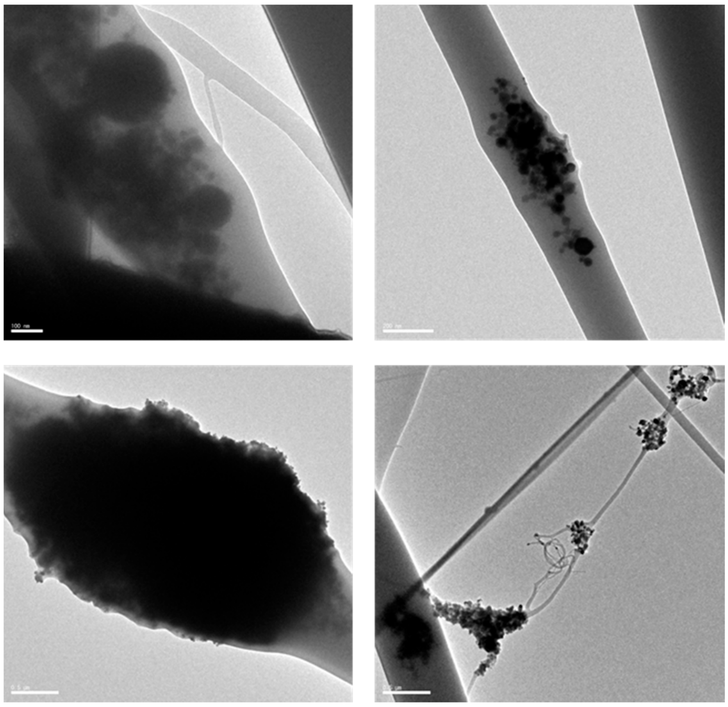

Figure S5. TEM observation of fiber with 60 wt % of MNPs. A scale bar is 500 nm.

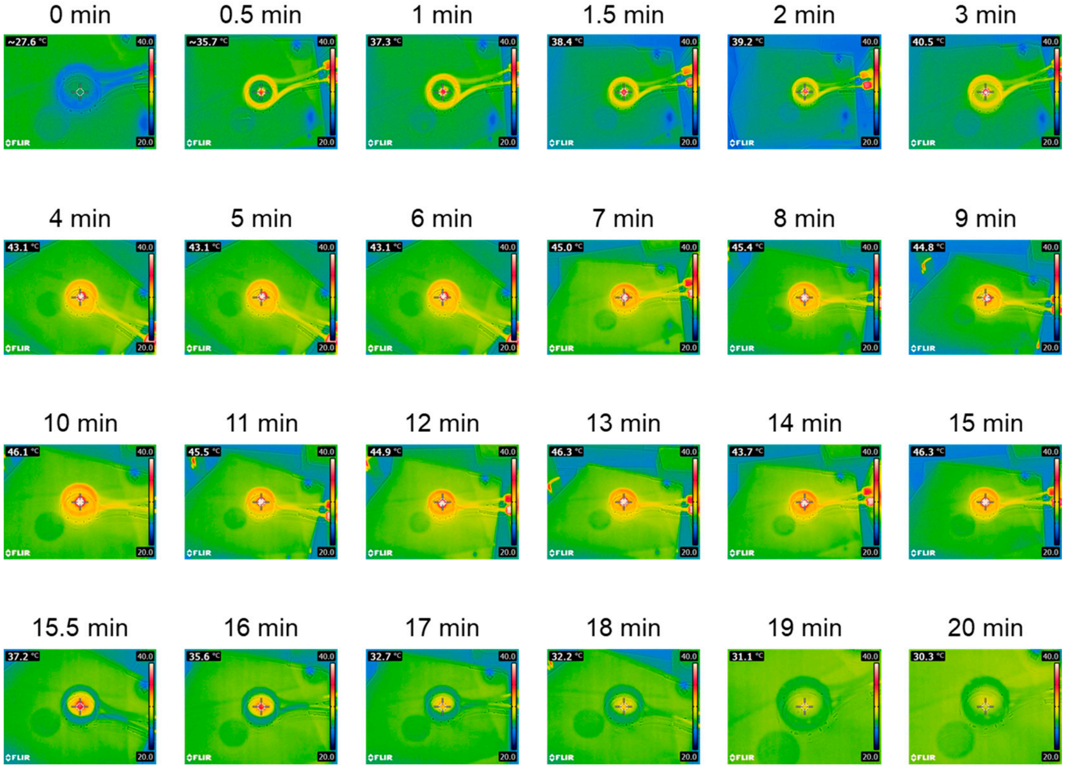

Figure S6. IR thermal images of the MNPs-incorporated fiber mesh in AMF. The AMF was turned on at time 0-min and turned off at time 15-min.

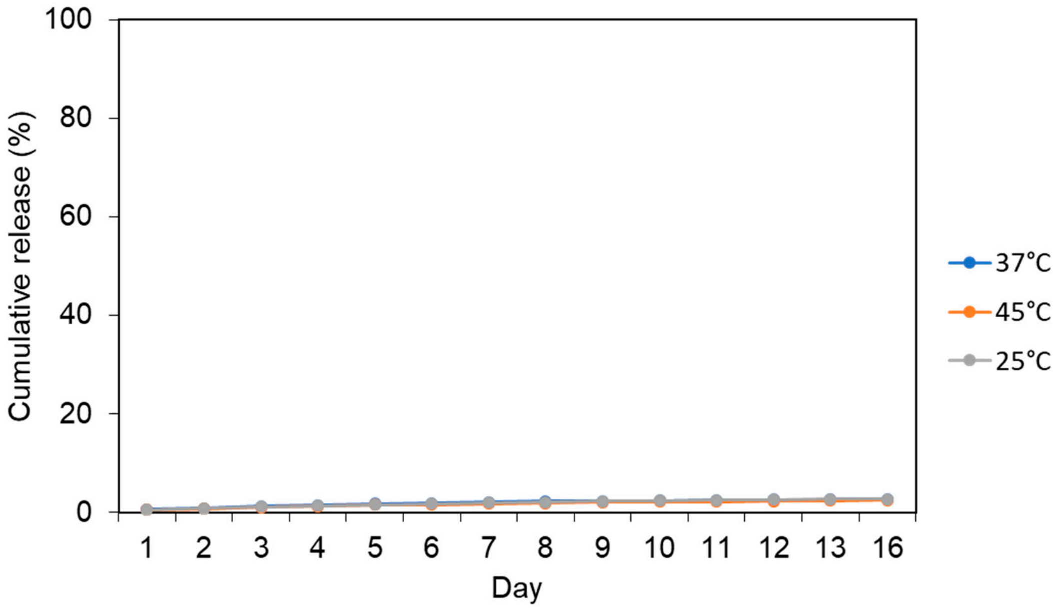

Figure S7. Long term drug release from the fiber mesh at 25 °C, 37 °C, and 43 °C.
